# Investigation of the Frequency Shift of a SAD Circuit Loop and the Internal Micro-Cantilever in a Gas Sensor

**DOI:** 10.3390/s100707044

**Published:** 2010-07-23

**Authors:** Liu Guan, Jiahao Zhao, Shijie Yu, Peng Li, Zheng You

**Affiliations:** Department of Precision Instruments and Mechanics, Tsinghua University, Beijing 100084, China; E-Mails: guanl04@mails.tsinghua.edu.cn (L.G.); yu-sj@tsinghua.edu.cn (S.Y.); yz-dpi@mail.tsinghua.edu.cn (Z.Y.)

**Keywords:** micro-cantilever, gas sensor, frequency shift, oscillator loop

## Abstract

Micro-cantilever sensors for mass detection using resonance frequency have attracted considerable attention over the last decade in the field of gas sensing. For such a sensing system, an oscillator circuit loop is conventionally used to actuate the micro-cantilever, and trace the frequency shifts. In this paper, gas experiments are introduced to investigate the mechanical resonance frequency shifts of the micro-cantilever within the circuit loop(mechanical resonance frequency, MRF) and resonating frequency shifts of the electric signal in the oscillator circuit (system working frequency, SWF). A silicon beam with a piezoelectric zinc oxide layer is employed in the experiment, and a Self-Actuating-Detecting (SAD) circuit loop is built to drive the micro-cantilever and to follow the frequency shifts. The differences between the two resonating frequencies and their shifts are discussed and analyzed, and a coefficient *α* related to the two frequency shifts is confirmed.

## 1. Introduction

Micromachined resonant devices are attracting increasing interest in the field of chemical sensor applications, due to some of their interesting properties such as sensitivity, compactness and low energy consumption [1]. Helped by an actuating and detecting circuit loop, the working frequency of the MEMS resonant devices can be followed automatically, and thus the analyte quantity can be determined [2].

In the study of Zhou *et al.* of Tsinghua University, a gas sensor based on a MEMS cantilever was introduced [3–4]. In their work, a piezoelectric micro-cantilever was used, with a sensitive layer deposited on the tip to adsorb gas molecules. The adsorption modifies the mechanical properties of the structure, and therefore the resonance frequency *f*_0_ shifts accordingly [5,6]. As the micro-cantilever has no detection structure, a self-actuating and detecting (SAD) circuit loop was designed specifically for this single-port device. The SAD circuit loop drives the micro-cantilever and traces the working frequency *f*_r_ of the circuit. The details of the SAD circuit loop will be introduced in Section 2.1.

Most of the actual studies follow the same pattern as presented previously [7–14]: on exposure to an analyte vapor, the additional mass loading of the polymer layer decreases the mechanical resonance frequency (MRF) of the micro-cantilever. This MRF shift, noted Δ*f*_0_, should be proportional to the mass loading Δ*m*, and thus proportional to the change in the gas concentration Δ*c* [1]. Nevertheless, in real world applications, as MRF usually is not available in operation, the system working frequency (SWF) shift of the actuating and detecting circuit loop, noted as Δ*f*_r_ in this study, is measured instead. The MRF shift and SWF shift are not actually identical. Li *et al.* first referred to the difference between the MRF shift and SWF shift, in a simulation approach [4].

An investigation is performed in this paper to demonstrate the existence of a coefficient *α*, which makes Δ*f*_r_ = *α*·Δ*f*_0_. The experimental investigation consists of an open-loop test, where the MRF shift Δ*f*_0_ is measured, and a close-loop test, where the SWF shift Δ*f*_r_ is recorded. The origin of this coefficient is also discussed. The results indicate that the conventional usage of SWF shift as a measurement of gas concentration is not appropriate, as the SWF shift is not identical to MRF shift. Instead, SWF shift measured should be revised by a coefficient to approach the MRF shift. The further discussion in this work also proves that this finding can be applied to all the sensing system using an actuating-detecting oscillator loop.

## 2. Experimental Details

### 2.1. Brief Description of the SAD Circuit Loop

With a presumption of small deformation, an electrical model of a piezoelectric micro-cantilever is developed [3].

The mechanical function analogy of the electrical components is presented in Equation (1). Where *m_effect_* is the effective mass of the micro-cantilever, *k* is the spring constant, *c_0_* is the static capacitance of the piezoelectric layer, *ξ* is the damping coefficient. The *k*_1_, *k*_2_ are proportionality constants:

(1) $$\left\{ \begin{array}{l} C1=k_{1}k_{2}\\ L1=\frac{m_{\mathrm{effect}}}{{\mathrm{kk}}_{1}k_{2}}\\ R1=\frac{2\xi \sqrt{{\mathrm{km}}_{\mathrm{effect}}}}{{\mathrm{kk}}_{1}k_{2}}\\ C0=c_{0}-k_{1}k_{2} \end{array}\right.$$

**Figure 2.**
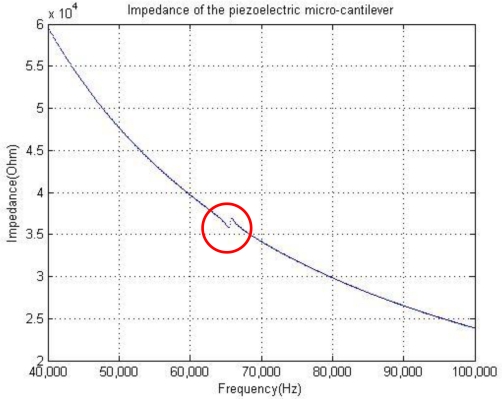
Impedance of the piezoelectric micro-cantilever.

Therefore, the electrical components in the model can be determined by the mechanical properties of the beam, among which *C*1 and *C*0 can be seen as a constant, *R*1 and *L*1 will vary with the frequencies. Furthermore, the impedance of the micro-cantilever can be determined, as shown in Figure 2. As the impedance of *C*0 is much smaller than that of *C*1-*R*1-*L*1 branch, the impedance of the micro-cantilever is rather determined by that of *C*0, and has only a local minimum at the MRF *f*_0_. This means the micro-cantilever does not have a good capability of selecting the resonance frequency.

**Figure 3.**
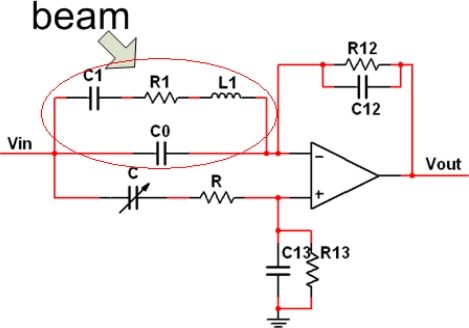
Frequency selection network module.

Therefore, for ameliorating the frequency selection performance of the beam, a frequency selection network module is designed. The constant capacitor *C*0 in the electrical model was compensated by an external tunable capacitor. In an ideal situation, the output of the network should perform as a band-pass filter, having maximum amplitude at the mechanical resonance frequency (see Figure 3).

**Figure 4.**
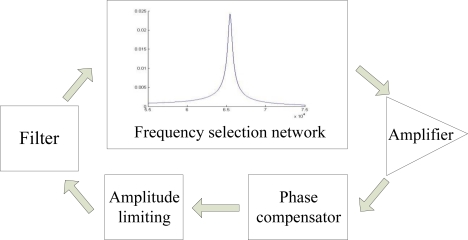
Schematic illustration of a SAD circuit.

Figure 4 shows the block diagram of the SAD circuit loop. The micro-cantilever is integrated in the module of frequency selection network, which constitutes the frequency determining unit of the oscillator circuit, and serves like a band-pass filter with a central frequency equal to *f*_0_. The output signal of the frequency selection network is amplified and then adjusted by the following phase compensator. An amplitude limiting module is used to control the amplitude of the signal. Finally, as in reality the output of frequency selection network cannot be reduced to zero at frequencies far away from *f*_0_, a band-pass filter is needed, to reduce the secondary mode oscillation of the beam.

Suppose the gain of the frequency selection network is *A*, and its phase-frequency curve is *φ_a_*, the rest of the SAD circuit loop is considered as a feedback module, with a gain of *F* and phase-frequency curve *φ_b_*. When the SAD circuit loop working in a close-loop mode, the Barkhausen conditions should be met [15]:

(2) $$\begin{array}{l} \left|A(f_{r})F(f_{r})\right|=1\\ \varphi_{a}(f_{r})+\varphi_{b}(f_{r})=2\pi \end{array}$$

Generally, the amplitude condition in (2) is not a crucial one, as the non-linearity in the feedback module makes the amplitude of the signal always satisfy the first equation. Therefore, the working frequency is rather determined by the second Equation of (2), the phase condition.

**Figure 5.**
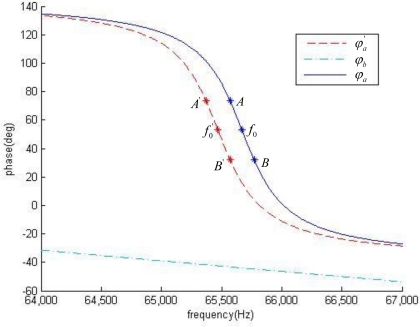
Simulation of phase-frequency curves of the SAD circuit loop, with AB and A’B’ the linear interval. Where *f*_0_ = 65,670 Hz and Δ*f*_0_ = 200 Hz.

Figure 5 illustrates the phase-frequency curves for frequency selection network as well as the feedback module. As seen from the figure, the feedback module shows a good linearity in its phase-frequency curves. And in a neighborhood of the mechanical resonance frequency, at least in the interval of *f*_0_ ± 100 Hz, the frequency selection network also shows a good linearity. After mass loading, mechanical resonance frequency decreases, and *φ_a_* (*f*) simply has a parallel shift of Δ*f*_0_, to *φ_a_* (*f*).

### 2.2. Analyte Concentration Controlling

The sensing system is mounted in a glass canister, with a thin glass slide as top window. Drops of liquid ethanol were jetted by a transfer pipette through this window to provide defined vapor concentrations to the canister. Suppose the volume of the canister is *V_vessel_*, and liquid ethanol with a volume *V_analyte_* is jetted into the canister. When volatilized completely, the concentration of ethanol vapor follows Equations (3) and (4) where *M* is the molecular weight of the analyte, and *P* is the ambient atmospheric pressure:

(3) $$C_{\left\{\mathrm{mg}/m^{3}\right\}}=\frac{\rho_{\mathrm{analyte}}V_{\mathrm{analyte}}}{V_{\mathrm{vessel}}}$$

(4) $$C_{\left\{\mathrm{ppm}\right\}}=\frac{C_{\left\{\mathrm{mg}/m^{3}\right\}}}{\frac{M}{22.4}\cdot \frac{273}{273+T}\cdot \frac{P}{1011325}}$$

For the experiments, we calculate the saturated concentration of ethanol vapor, known the ambient temperature and pressure of atmosphere. Then, an appropriate volume *V_analyte_* is chosen, to make sure that with six liquid injections, the concentration of the ethanol vapor in the canister increases at a constant pace and is finally below the saturated level. The open-loop test and the close-loop one are performed in the same canister, with the same ambient conditions. The transfer pipette has a precision of measurement of 0.3%, which ensures the coherence of the two tests, and the comparability of the results.

### 2.3. Open-Loop Tests

**Figure 6.**
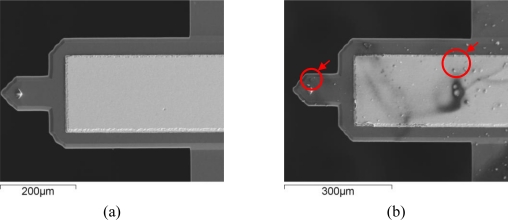
Scanning electron micrographs of the micro-cantilever: (a) Before the deposition of PEO sensitive material. (b) After the deposition of PEO sensitive material.

An Open-loop test records the variation of mechanical resonance frequency *f*_0_ of the micro-cantilever. The micro-cantilever used in this work is a probe from a commercial Atomic Force Microscopy (AFM) system supplied by the Veeco^®^ Company. It consists of a micro-machined silicon beam with a piezoelectric zinc oxide layer. The MRF of the AFM cantilever is 50,000 Hz, according to the specificationz and is measured at 66,640 Hz. Polyethyleneoxide (PEO) was chosen as the sensitive material, for its good sensitivity and selectivity to ethanol vapor [16]. An aqueous solution of PEO was jetted on top of the cantilever. After the evaporation of the solvent, the tip of the cantilever was covered with a layer of sensitive material in the form of granule (Figure 6), and the MRF of the cantilever decreases to 65,670 Hz accordingly.

**Figure 7.**
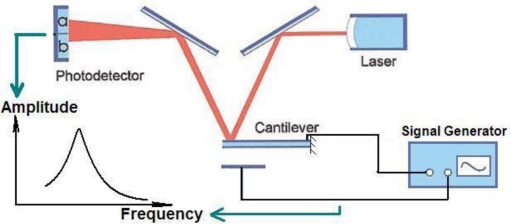
Schematic drawing of the experimental setup of the open-loop tests [3].

The open-loop test instruments system is illustrated by Figure 7. A signal generator provides an actuating signal, and the laser beam was focused on the free-moving end of the coated cantilever. The laser beam deflection signal was captured by a photodetector, and the amplitude of the oscillation was read out from the screen. By sweeping the actuating signal near the mechanical resonance frequency, *f*_0_ can be detected. The Q-factor of the coated probe is near 130 at ambient conditions of standard atmospheric pressure and 293 K temperature.

### 2.4. Close-Loop Tests

In a close-loop experiment, the system working frequency *f*_r_ of the SAD circuit loop was detected. Schematic diagram of the test can is illustrated in Figure 4. The resolving power of the tests is approximately 0.5 Hz. To ensure that the SAD circuit loop works in its closed-loop mode, the following two adjustments should be made. Firstly, the tunable capacitor in the frequency selection network should be adjusted to compensate *C*0, that is to say, at a frequency *f* ≠ *f*_0_, the output of the frequency selection network is equal to 0. Secondly, connect the frequency selection network with the feedback module, and adjust the phase compensator, to meet the phase condition in the Barkhausen conditions.

## 3. Results and Discussion

### 3.1. Initial Offsets

**Figure 8.**
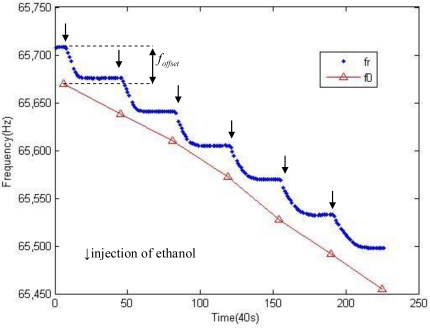
Typical raw data of the frequency shift of a SAD circuit loop and the internal micro-cantilever. To improve the visibility of the data points, the data are sampled with a time step of 40 seconds. The abscissa is time and each unit represents 40 seconds.

A pair of experimental datapoints can be observed in Figure 8. The liquid ethanol was jetted every 30 minutes in both of the tests. The set of triangle symbols represents the mechanical resonance frequency in the open-loop test. The step-form points record the working frequency of the SAD circuit loop as a function of time. As the volatilization takes place, the working frequency of the circuit loop decreases and at the end of the volatilization, the working frequency stabilizes gradually. As mentioned at the beginning, it is the coefficient *α* between the SWF shift Δ*f*_r_ and the MRF shift Δ*f*_0_ what we exploit. It is easy to notice that in this result, the initial frequency, which corresponds to a vapor concentration of 0, is different. Such an offset of the initial frequency is caused the by the offset of the initial phase. During the adjustment of the phase compensator before a close-loop test, an error of operation is inevitable as the signal superposition is depending on subjective judgment. Therefore, an offset of initial phase was introduced, noted as *φ_offset_*. The frequency offset *f_offset_* is thereby created. The frequency offset is 38 Hz in this experiment.

As *φ_offset_* is the phase offset at *f* = *f*_0_ in an open-loop adjustment, from (2) we have:

(5) $$\varphi_{a}(f_{0})+\varphi_{b}(f_{0})=2\pi +\varphi_{\mathrm{offset}}$$

When the system works in a close-loop mode, we also have:

(6) $$\varphi_{a}(f_{0}+f_{\mathrm{offset}})+\varphi_{b}(f_{0}+f_{\mathrm{offset}})=2\pi$$

Developing (6) at *f* = *f*_0_, using a Taylor development:

$$\begin{array}{l} \varphi_{a}(f_{0})+\frac{\partial \varphi_{a}(f_{0})}{\partial f}f_{\mathrm{offset}}+\frac{1}{2!}\frac{\partial \varphi_{a}^{2}(f_{0})}{\partial f^{2}}f_{\mathrm{offset}}^{2}+...\\ +\varphi_{b}(f_{0})+\frac{\partial \varphi_{b}(f_{0})}{\partial f}f_{\mathrm{offset}}+\frac{1}{2!}\frac{\partial \varphi_{b}^{2}(f_{0})}{\partial f^{2}}f_{\mathrm{offset}}^{2}+...=2\pi \end{array}$$

As the linearity shown in Figure 4, in the interval of this development, the high order derivatives are far less than the first order derivative. Thus:

(7) $$\varphi_{a}(f_{0})+\frac{\partial \varphi_{a}(f_{0})}{\partial f}f_{\mathrm{offset}}+\varphi_{b}(f_{0})+\frac{\partial \varphi_{b}(f_{0})}{\partial f}f_{\mathrm{offset}}=2\pi$$

From (5) and (7), we have:

(8) $$f_{\mathrm{offset}}=\frac{\varphi_{\mathrm{offset}}}{\frac{\partial \varphi_{a}(f_{0})}{\partial f}+\frac{\partial \varphi_{b}(f_{0})}{\partial f}}$$

It is seen from (8) how *φ_offset_* causes *f_offset_*, the offset of the initial frequency in experimental results. The literature expression of *α* later on will show that this coefficient will not be affected by the initial frequency offset.

### 3.2. Differences between MRF Shifts and SWF Shifts

**Figure 9.**
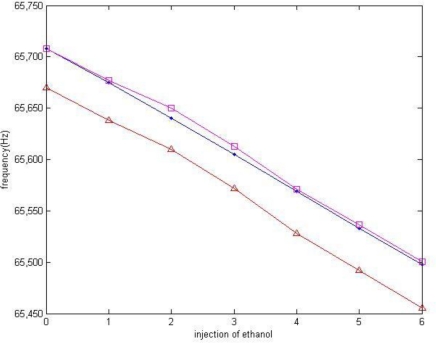
Frequency shift in response to the injection of ethanol. **(a)** MRF of the micro-cantilever in open-loop test [ 
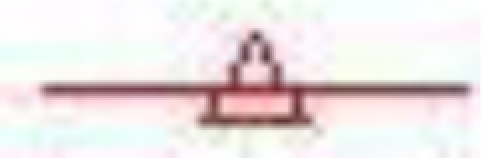
]. **(b)** SWF in close-loop test [ 
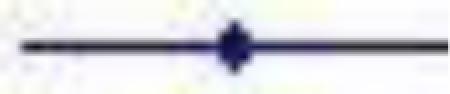
]. **(c)** Simulation results of SWF [ 
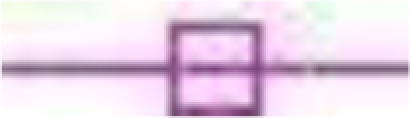
].

Figure 9 shows the MRF and the SWF as a function of gas concentration. After six sequential ethanol injections of equal quantity, the MRF decreases from 65,670 Hz to 65,455 Hz, and the SWF decreases from 65,708 Hz to 65,498 Hz. A result of simulation of SWF calculated from the MRF measured is also presented for comparison.

It is the ratio of the slope of two experimental curves that represents the coefficient *α*. The linear expression is obtained by interpolating the curves using the least-square principle. As the close-loop has a higher resolution power, its data shows a better correlation:

$$\begin{array}{c} f_{0}:\;\;y=-36.393x+65676,\;R^{2}=0.9966\\ f_{r}:\;\;y=-35.179x+65710,\;R^{2}=0.9999\\ f_{r-simulate}:\;\;y=-35.089x+65710,\;R^{2}=0.9969 \end{array}$$

Both experimental result and simulation result demonstrate the existence of a coefficient *α* which makes Δ*f*_r_ = *α*·Δ*f*_0_. The value of *α* is also calculated: *α_real_ =* 0.9666, *α_simulate_* = 0.9642.

We are going to explore this a little bit further. As a frequency offset *f_offset_* is observed, we are going to demonstrate it has no influence to *α*. Suppose that the SWF of the SAD circuit loop decreases from *f*_r_ to $f_{r}^{\prime }$, when the MRF decreases form *f*_0_ to $f_{0}^{\prime }$. Transform the definition $f_{r}-f_{r}^{\prime }=\alpha (f_{0}-f_{0}^{\prime })$ to:

(9) $$(1-\alpha )(f_{0}-f_{0}^{\prime })=f_{\mathrm{offset}}^{\prime }-f_{\mathrm{offset}}$$

After mass loading, at the new resonance frequency $f_{0}^{\prime }$, a new phase offset $\varphi_{\mathrm{offset}}^{\prime }$ exists:

(10) $$\varphi_{a}^{\prime }(f_{0}^{\prime })+\varphi_{b}(f_{0}^{\prime })=2\pi +\varphi_{\mathrm{offset}}^{\prime }$$

From the discussion of Figure 4, there is:

(11) $$\varphi_{a}(f)=\varphi_{a}^{\prime }(f-\Delta f_{0})$$

A deduction follows the routine of (8), we have:

(12) $$f_{\mathrm{offset}}^{\prime }=\frac{\varphi_{\mathrm{offset}}^{\prime }}{\frac{\partial \varphi_{a}^{\prime }(f_{0}^{\prime })}{\partial f}+\frac{\partial \varphi_{b}(f_{0}^{\prime })}{\partial f}}$$

For the first term of the denominator, use (11), we have:

(13) $$\frac{\partial \varphi_{a}^{\prime }(f_{0}^{\prime })}{\partial f}=\frac{\partial \varphi_{a}^{\prime }(f_{0}-\Delta f_{0})}{\partial f}=\frac{\partial \varphi_{a}(f_{0})}{\partial f}$$

For the second term of the denominator, develop $\frac{{\partial \varphi }_{b}(f_{0})}{\partial f}$ using Taylor development at $f_{0}^{\prime }$:

(14) $$\frac{{\partial \varphi }_{b}(f_{0})}{\partial f}=\frac{{\partial \varphi }_{b}(f_{0}^{\prime })}{\partial f}+\frac{1}{2!}\frac{\partial^{2}\varphi_{b}(f_{0}^{\prime })}{\partial f^{2}}\Delta f_{0}+\frac{1}{3!}\frac{\partial^{3}\varphi_{b}(f_{0}^{\prime })}{\partial f^{3}}\Delta f_{0}^{2}+...$$

Notice the linearity of *φ_b_*(*f*), we have:

(15) $$\frac{\partial \varphi_{b}(f_{0}^{\prime })}{\partial f}=\frac{\partial \varphi_{b}(f_{0})}{\partial f}$$

Substitute the denominator of (12) by (13) and (15), using (8), we have:

(16) $$f_{\mathrm{offset}}^{\prime }-f_{\mathrm{offset}}=\frac{\varphi_{\mathrm{offset}}^{\prime }-\varphi_{\mathrm{offset}}}{\frac{\partial \varphi_{a}(f_{0})}{\partial f}+\frac{\partial \varphi_{b}(f_{0})}{\partial f}}$$

Using (5) and (10), we have:

(17) $$\varphi_{\mathrm{offset}}^{\prime }-\varphi_{\mathrm{offset}}=\frac{\partial \varphi_{b}(f_{0})}{\partial f}\Delta f_{0}$$

Thus:

(18) $$f_{\mathrm{offset}}^{\prime }-f_{\mathrm{offset}}=\frac{\frac{\partial \varphi_{b}(f_{0})}{\partial f}}{\frac{\partial \varphi_{a}(f_{0})}{\partial f}+\frac{\partial \varphi_{b}(f_{0})}{\partial f}}\Delta f_{0}$$

From (18) *α* can be written as:

(19) $$\alpha =\frac{\frac{{\partial \varphi }_{a}(f_{0})}{\partial f}}{\frac{\partial \varphi_{a}(f_{0})}{\partial f}+\frac{{\partial \varphi }_{b}(f_{0})}{\partial f}}$$

The literature expression shows that *α* is independent with *φ_offset_*. It is the phase-frequency curve of frequency selection network and of feedback module that determine the coefficient. As shown in Figure 5, at the point of *f*_0_, the slope of the phase-frequency curve *φ_a_* (*f*) is much larger than that of *φ_b_* (*f*), thereby the coefficient *α* has a value close to 1.

**Figure 10.**
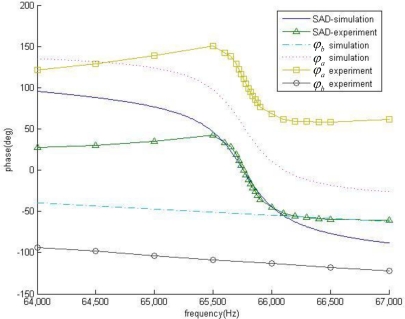
Experimental phase-frequency curves in compare with simulation results at MRF *f*_0_ = 65,670 Hz and SWF *f*_r_ = 65,708 Hz. (a) Simulation results for SAD[ 
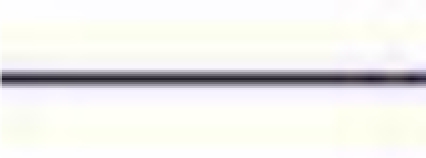
], feedback module[ 
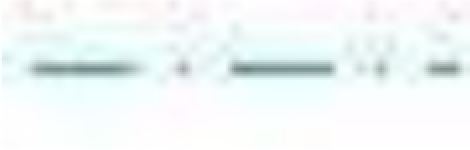
] and frequency selection network[ 
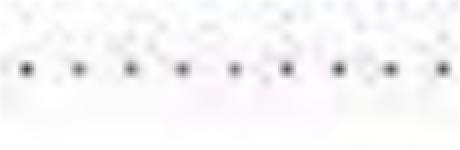
]. (b) Experimental measurements of SAD[ 
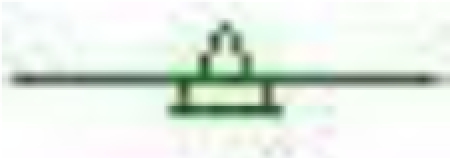
], feedback module[ 
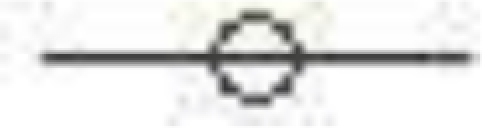
] and frequency selection network[ 
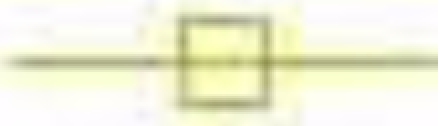
].

To calculate the coefficient *α* from Equation (19), we draw the phase-frequency curves in Figure 10, where the experimental data is presented together with the simulation one. For the frequency selection network, both the results from experiment and simulation show a good linearity at the neighborhood of *f*_0_, whereas at the point far away from *f*_0_, the slopes of the two curves deviate. The deviation is likely to be a result of non-ideal properties of the micro-cantilever. As for the feedback module, both two curves show a good linearity, and an approximate slope.

Based on literature result in (19), the coefficient *α* can be determined from the slopes of the phase-frequency curves: *α_real_* = 0.9659, *α_simulate_* = 0.9640. These results confirm our findings in gas experiments. Furthermore, as the coefficient *α* is related to the phase-frequency curves of the frequency selection network and of the feedback module, it is a typical value for each realization of a SAD circuit loop.

## 4. Conclusions

Gas experiments have been performed on a MEMS gas sensing system. The system is based on a silicon beam with a piezoelectric zinc oxide layer, and a SAD circuit loop is built to actuate the cantilever and detect its frequency shifts. The experimental results confirm, as predicted by another paper, the existence of a coefficient *α*, relating the mechanical resonance frequency shift and system working frequency shift. It was also discussed the relation between this coefficient and the phase-frequency curves of the SAD circuit loop.

As analyzed in this paper, the mass loading of the micro-cantilever modifies its MRF as well as the phase-frequency curves of the SAD circuit loop, and therefore the SWF shifts to meet the phase condition of Formula (2) so that the SAD circuit loop could continue its close-loop working. This paper shows that the SWF shift observed in operations is not an appropriate indicator of the gas concentration. To characterize the concentration of analyte, the SWF shift should be revised with a coefficient, to trace the MRF shift. And as such a coefficient is derived from the Barkhausen conditions, it should be taken into account in all kinds of sensing system working with an actuating-detecting circuit loop.

## Figures and Tables

**Figure 1.**
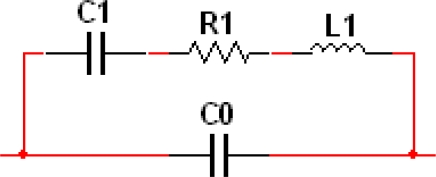
Electrical model of piezoelectric micro-cantilever.
